# Supplementary material for: Predicting migration routes for three species of migratory bats using species distribution models
Source: PeerJ. 2021 Apr 16;9:e11177. doi: 10.7717/peerj.11177 (PMC8054759; doi:10.7717/peerj.11177)
Supplement: Supplemental Information 1 — More in-depth description of methods used in analyses. [file peerj-09-11177-s001.docx]

**Methods**

*Occurrence data from GBIF*

Figure 1 shows an overview of the steps involved in data collection and analysis. To begin, all available occurrence data were downloaded for *L. borealis, L. cinereus*, and *L. noctivagans* through the GBIF data portal (<http://www.gbif.org>) on 11 March 2019 using only ‘Preserved Specimens’, ‘Human Observations’, and ‘Material Sample’ keywords from the past 50 years (<https://doi.org/10.15468/dl.dpiwzi>, <https://doi.org/10.15468/dl.irfol0>, and <https://doi.org/10.15468/dl.viiyt5>, respectively). This 50-year time period was selected as it allows for more confidence in the call of a species and its locality. All downloaded records were then screened using several filters (described below) as recommended by others (Feeley & Silman, 2011; Carstens, Morales, Field, & Pelletier, 2018). Specifically, we only included records with geographic collection coordinates and ‘without known coordinate issues’ or coordinates that were ‘not suspect’. Any records with obvious geographic errors (e.g. those occurring in bodies of water or those occurring in regions far beyond current range), occurrences outside of North America, and geographic outliers were removed. Lastly, we excluded any records that did not include a date of collection. Once data sets were filtered using these criteria, we corrected for over sampling within a 1^o^ region following guidelines given by Hijmans and Elith (2017). In brief, we created a grid of 1^o^ resolution and subsampled our occurrence data to one occurrence per grid cell. This was done to reduce the possibility of sampling bias in our data.

*Predictor environmental variables*

WorldClim version 2 monthly climatic data were used at 2.5-minute resolution (Fick & Hijmans, 2017) for our species distribution models and included the following variables: precipitation, solar radiation, average temperature, maximum temperature, minimum temperature, vapor pressure, and wind speed (downloaded on 03 June 2019 from worldclim.org). Additionally, elevation maps (Tachikawa et al., 2011; 11 March 2019), and the human influence index (WCS, 2005; 11 March 2019) for North America were also downloaded as Jung and Threlfall (2016) showed a negative response to urbanization in the Americas in insectivorous bats in the family Vespertilionidae. Human influence was determined by combining population density, human land use and infrastructure, and human access (WCS, 2005). Following Hayes et al. (2015), we also included MODIS Normalized Difference Vegetative Index (Didan, Munoz, Solano, & Huete, 2015) and Global Tree Coverage 2010 (Hansen et al., 2013) as metrics of seasonality and leaf growth, which could impact prey abundance, and be a metric of available roost sites in trees, downloaded on 04 June 2019 and 05 June 2019, respectively. Prior to final selection of predictor variables, correlations between each possible pair of predictor variables was determined and one variable from each pair that was strongly correlated with the other was removed (r > 0.8; Mateo, Vanderpoorten, Munoz, Laenen, & Desamore, 2013). Any removal of a variable was determined based on biological relevance and previous uses in literature.

*Species distribution modeling*

Species distribution models were generated for each species using five different methods: four specific model algorithms and an ensemble approach (see below). Specific algorithms included: generalized linear model (GLM), BIOCLIM model (BC), random forest (RF), maximum entropy (MaxEnt; Phillips, Dudik, & Schapire, 2017). Of these, MaxEnt is the most widely used method, especially when only single models are used. However, its accuracy has been questioned when compared to many other models, highlighting a need to test multiple models (Qiao, Soberon, & Peterson, 2015). BIOCLIM has the advantage of only requiring presence data, but the comparison of new sites based on habitat suitability but can underperform compared to other methods (Booth, Nix, Busby, & Hutchinson, 2014). On the other hand, models like GLM need some type of absence data (approximated here) which can be difficult to attain for analysis but have a better ability to realistically model ecological relationships (Austin, 2002). Random forest also requires absence points during its modeling, and while statistically rigorous, this method can tend to overfit the data (Qiao et al., 2015). MaxEnt has an advantage through being designed for use with pseudoabsence points and has a long history of success of use for modeling, but recent analysis has shown some potential biases that are difficult to address, such as sampling bias (Syfert, Smith, & Coomes, 2013). While many other methods are available, these methods are commonly used and have shown to be effective in broadly distributed taxa (Qiao et al., 2015).

Finally, another approach to account for any single problem in a model is ensemble modeling which incorporates the best features of each model while accounting for some of the weaknesses (Araújo & New, 2006). This approach is becoming more common for use in SDMs (Razgour, Rebelo, Di Febbraro, & Russo, 2016) and may increase the accuracy of the final model used (Marmion, Parviainen, Luoto, Heikkinen, & Thuiller, 2009). This is especially true when using weighted averaging to account for any pitfalls that a single model may be displaying (Qiao et al., 2015). Due to this, the weight for each model was assessed on a species and month basis to ensure the best possible model performance.

All SDM analyses were carried out in R using the packages “randomForest” (Liaw & Wiener, 2018), “raster” (Hijmans et al., 2019), “rgeos” (Bivand et al. 2019), “maptools” (Bivand et al. 2019), “dismo” (Hijmans, Phillips, Leathwick, Elith, & Hijmans, 2017), “sp” (Pebesma, Bivand, & Pebesma, 2012), “ecospat” (Di Cola et al. 2016), and “rJava” (Urbanek, 2019). We created 1000 pseudo absence points for each month from random points in the background layers and partitioned the model into testing (80%) and training data (20%) using the “kfold” function. Random pseudo absence points were selected to increase reliability of the output models (Barbet-Massin, Jiguet, Albert, & Thuiller, 2012). Each model produced an estimate of potential habitat suitability for each pixel, expressed continuously between 0 and 1. The exception was random forest as the output used was binary and so each pixel was coded as either 0 (not suitable) or 1 (suitable).

Each model was then assessed using two metrics calculated from a test dataset that was not used for generating the model: 1) Area under the ROC (receiver operating characteristic) curve (AUC) and 2) True Skill Statistic (TSS). While other metrics are available such as sensitivity (proportion of correctly predict presences), specificity (proportion of correctly predicted absences), and kappa (relating the accuracy of the model by accuracy expected to occur by chance), each have their downfalls. Sensitivity and specificity are accounted for in TSS and TSS is less impacted by prevalence (Allouche, Tsoar, & Kadmon, 2006). Additionally, kappa is also dependent on prevalence of occurrence points which can skew results. Consequently, TSS has been argued to be a better metric of performance due to is ability to correct for prevalence and keeping the advantages of kappa. AUC on the other hand is a more traditional metric of evaluating SDM models and is a measure of predictive model quality independent of applying a threshold (Beck, Böller, Erhardt, & Schwanghart, 2014).

For our ensemble model we used the AUC and TSS performance calculated above to weigh each individual model. These weight-transformed models were then averaged into a single SDM model. Following generation of our ensemble models, they were assessed using the same AUC and TSS metrics as outlined above and data points used for all other models for comparison to determine which model to use for further analysis. These layers were used to predict migratory pathways.

The importance of individual variables was assessed using different methods for each model. For RF we used the ‘importance’ function in the “randomForest” R package to measure the importance of a variable in a given model. With MaxEnt, variable importance was assessed using ‘var.importance’ function in “ENMeval” to determine the importance of each variable (Muscarella et al., 2014). For the GLM model, we used the ‘varImp’ function present in “caret” (Kuhn et al., 2019).

*Migratory pathways*

To identify migratory pathways using SDMs we used three complementary methods: circuit theory (McRae & Beier, 2007; Shah & McRae, 2008), 95^th^ percentile suitability (Poor, Loucks, Jakes, & Urban, 2012), and least cost path analyses (LCP; Howey, 2011). Since each of these methods have advantages and disadvantages, results from these three methods were compared to come up with a consensus definition of possible corridors (Bond, Bradley, Kiffner, Morrison, & Lee, 2017; Marrotte & Bowman 2017). While some have argued for selecting the single best hypothesized approach (Marrotte & Bowman 2017), as we do not know if these species follow linear features as has been observed in some species (Ahlén et al. 2009) or exhibit more erratic movements, we could not confidently select a singular approach. For circuit theory, the protocol of Burke, Frey, Ganguli, and Stoner (2019) was followed. In brief, we aggregated our winter month occurrences (December – February) into a single dataset and did the same for summer months (June-July), using Hayes et al., (2015) to determine the appropriate months for each season. As SDMs can be interpreted as conductance maps, we used an average of both spring and fall months (March, April, May; and August, September, October, respectively) to assess potential corridors between winter and summer occurrences. These time periods are based on previously published distributions of occurrences (Cryan 2003), previous SDM modeling (Hayes et al. 2015), wind farm fatality data (Arnett et al. 2008), radio telemetry (Walters et al. 2006), and acoustic data (Muthersbaugh et al. 2019). Using Circuitscape (Shah & McRae, 2008), we set our start (“source”) and end (“ground”) points based on the hypothesized direction of migration. To identify patterns of spring migration, we set our start as winter occurrences and end as summer points, with the Spring SDMs as the conductance raster; and summer as start and winter as end with Fall SDMs as the fall migration conductance raster; this was repeated for each species.

To use least cost path analysis to predict migratory pathways we used the R function ‘shortestPath’ implemented in ‘gdistance’ (Van Etten, 2017). The analysis was done iteratively between all points previously designated as “Winter” and “Summer” points for Circuitscape, and Spring/Fall conductance surfaces for cost determination. As single pathways are not informative for species-wide migratory pathways, we combined each least cost path to create a density of pathways. A high density of overlapping paths was used to identify a migratory pathway. Additionally, while we are unable to infer if a proposed path is true, we used Moran’s I (Moran, 1950) and Geary’s C (Geary, 1954) to quantify if these proposed pathways are positively clustered, as would be expected in a migratory corridor. We also quantified the distance traveled compared to straight-line distance to determine if the proposed pathways would be biologically relevant (i.e. if not following straight line, other factors influence where bats migrate through).

Next, binary rasters identifying potential migratory pathways using the 95^th^ percentile approach was generated to identify areas where bats are more likely to be concentrated compared to background (Poor et al., 2012). This was to identify areas where bats suitability is higher and therefore a potential migratory pathway. Finally, overlaps between Circuitscape, least cost path, and 95^th^ percentile approaches were identified to highlight locations where they agreed and those were assessed to be potential migratory pathways.

To ensure we are tracking migration and not simply sampling bias, a comparison between the results for migratory pathways above and those from two non-long-distance migratory species (*Myotis lucifugus* and *Eptesicus fuscus*) following the same methods above was carried out. If the pathways are similar to those from these two species it is possible that we are tracking the ability to capture bats during the winter instead of actual movement. On the other hand, if pathways are different, then it is more likely that we are identifying true pathways. Occurrence data for these additional species were collected from GBIF on 31 January 2020 (https://doi.org/10.15468/dl.fphagx) and filtered the same way as previous species, followed by SDM generation and pathway analysis following the same steps and procedures used for the three migratory species.

**References**

Allouche, O., Tsoar, A., & Kadmon, R. (2006). Assessing the accuracy of species distribution models: prevalence, kappa and the true skill statistic (TSS). *Journal of Applied Ecology*, *43*, 1223–1232.

Austin, M. (2007). Species distribution models and ecological theory: a critical assessment and some possible new approaches. *Ecological Modelling*, *200*, 1–19.

Barbet‐Massin, M., Jiguet, F., Albert, C. H., & Thuiller, W. (2012). Selecting pseudo‐absences for species distribution models: how, where and how many? *Methods in Cology and Evolution*, *3*, 327–338.

Beck, J., Böller, M., Erhardt, A., & Schwanghart, W. (2014). Spatial bias in the GBIF database and its effect on modeling species’ geographic distributions. *Ecological Informatics*, *19*, 10–15.

Booth, T. H., Nix, H. A., Busby, J. R., & Hutchinson, M. F. (2014). BIOCLIM: the first species distribution modelling package, its early applications and relevance to most current MAXENT studies. *Diversity and Distributions*, *20*, 1–9.

Lawson, C. R., Hodgson, J. A., Wilson, R. J., & Richards, S. A. (2014). Prevalence, thresholds and the performance of presence–absence models. *Methods in Ecology and Evolution*, *5*, 54–64.

Qiao, H., Soberon, J., & Peterson, A. T. (2015). No silver bullets in correlative ecological niche modeling: insights from no silver bullets in correlative ecological niche modelling: insights from testing among many potential algorithms for niche estimation. *Methods in Ecology and Evolution*, *6*, 1126–1136.

Syfert, M. M., Smith, M. J., & Coomes, D. A. (2013). The effects of sampling bias and model complexity on the predictive performance of MaxEnt species distribution models. *PLoS ONE*, *8*, e55158.
