# Supplementary figures and images for: Predicting migration routes for three species of migratory bats using species distribution models
Source: PeerJ. 2021 Apr 16;9:e11177. doi: 10.7717/peerj.11177 (PMC8054759; doi:10.7717/peerj.11177)

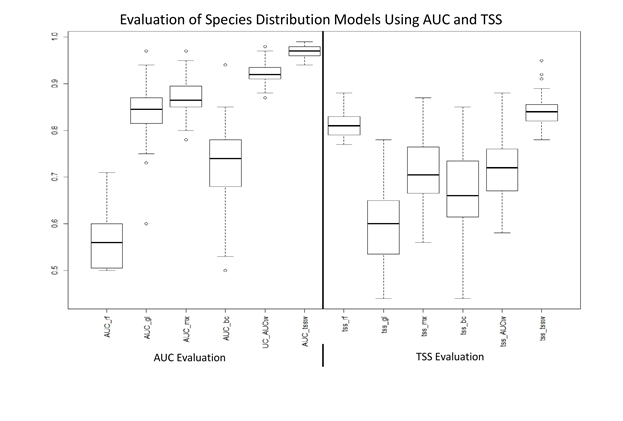

Supplement: Supplemental Information 2 — AUC and TSS values of model performance for each model (RF, GLM, MaxENT, and BC) and an ensemble model weighted by either AUC or TSS performance, in that order. Using both metrics, the TSS weighted ensemble model is seen as the best performing. [file peerj-09-11177-s002.png]

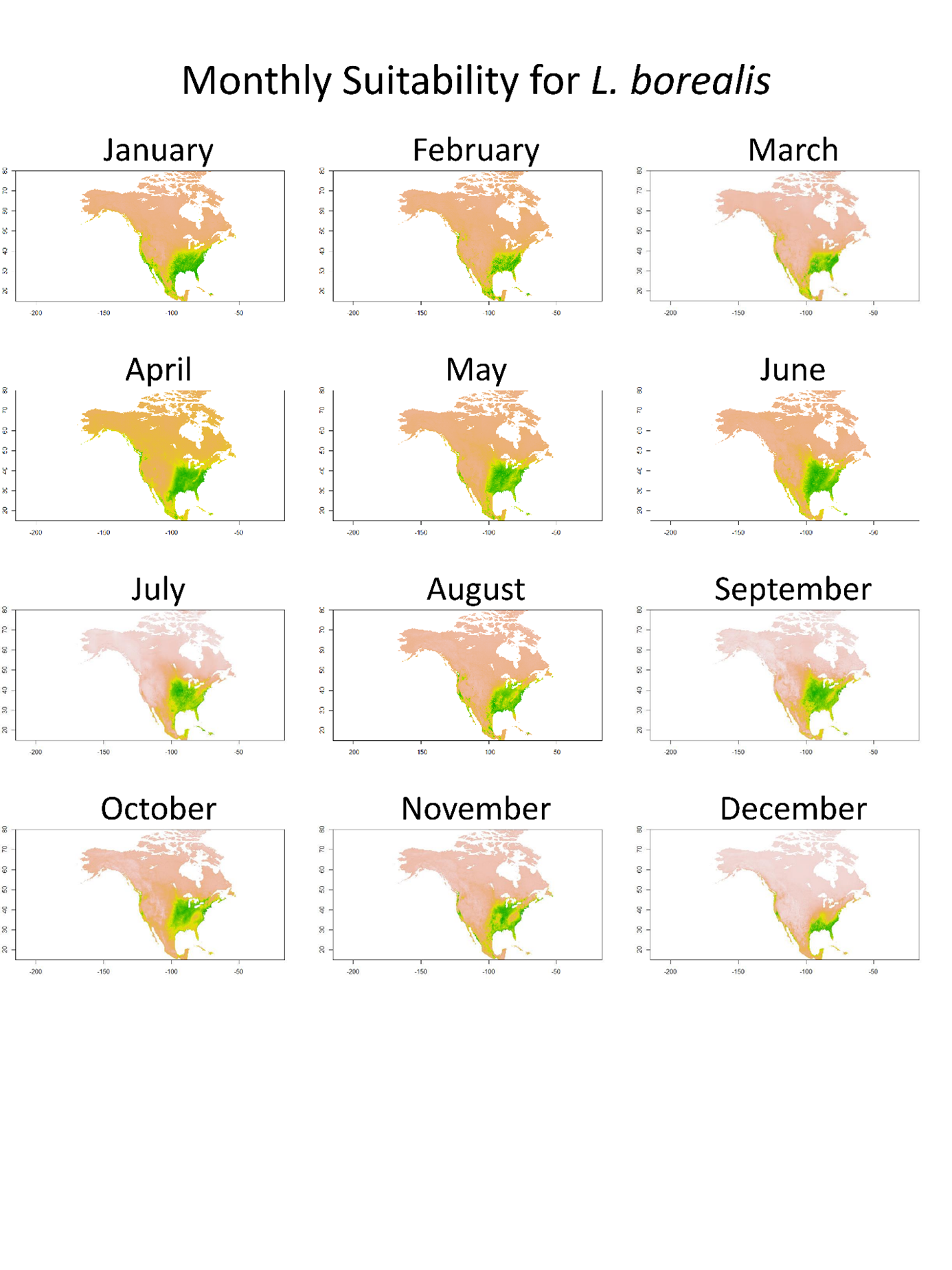

Supplement: Supplemental Information 3 — Monthly SDM from TSS weighted ensemble model for L. borealis across North America. Green equals higher suitability, while yellow/tan show lower suitability. [file peerj-09-11177-s003.png]

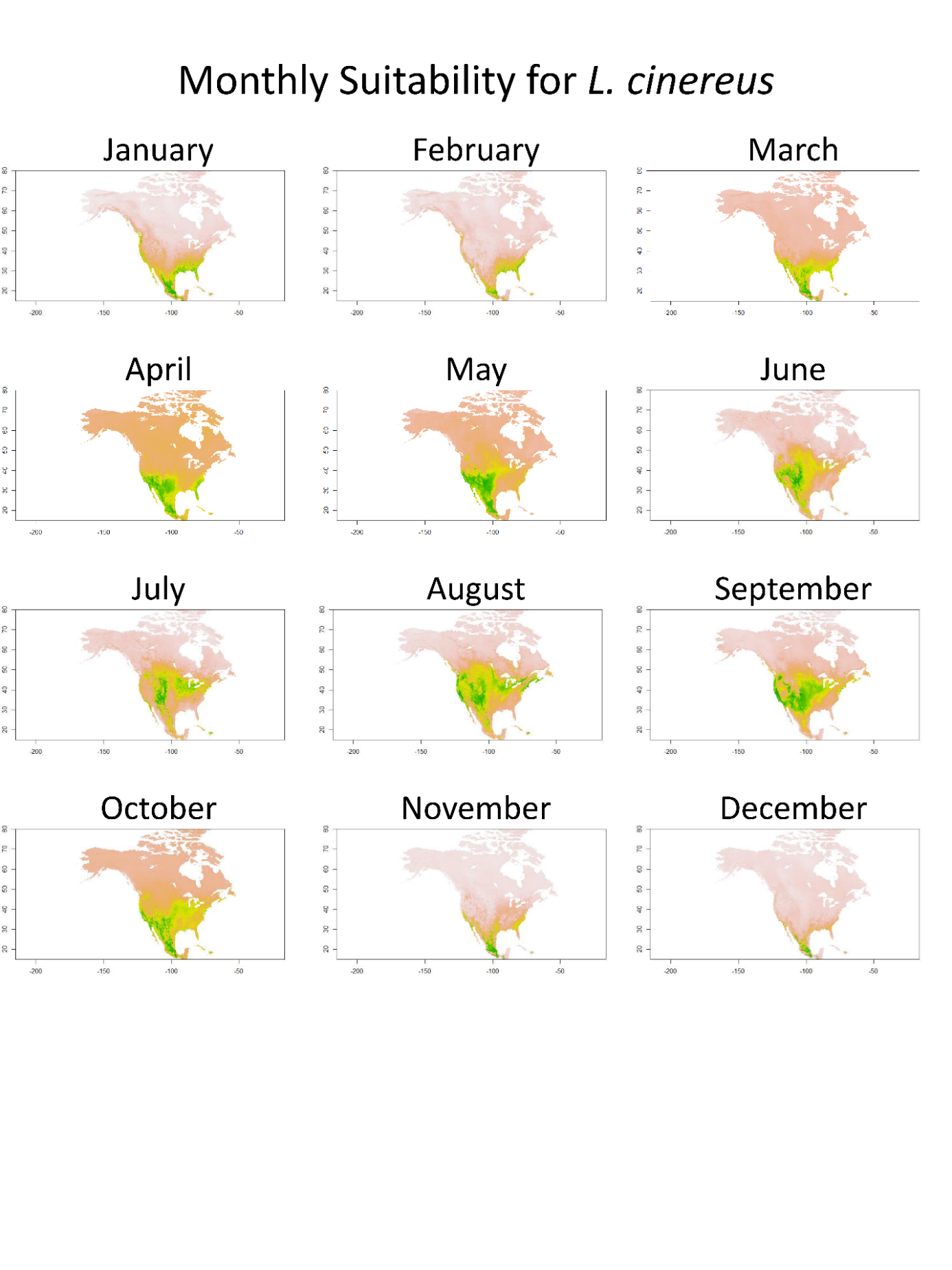

Supplement: Supplemental Information 4 — Monthly SDM from TSS weighted ensemble model for L. cinereus across North America. Green equals higher suitability, while yellow/tan show lower suitability. [file peerj-09-11177-s004.png]

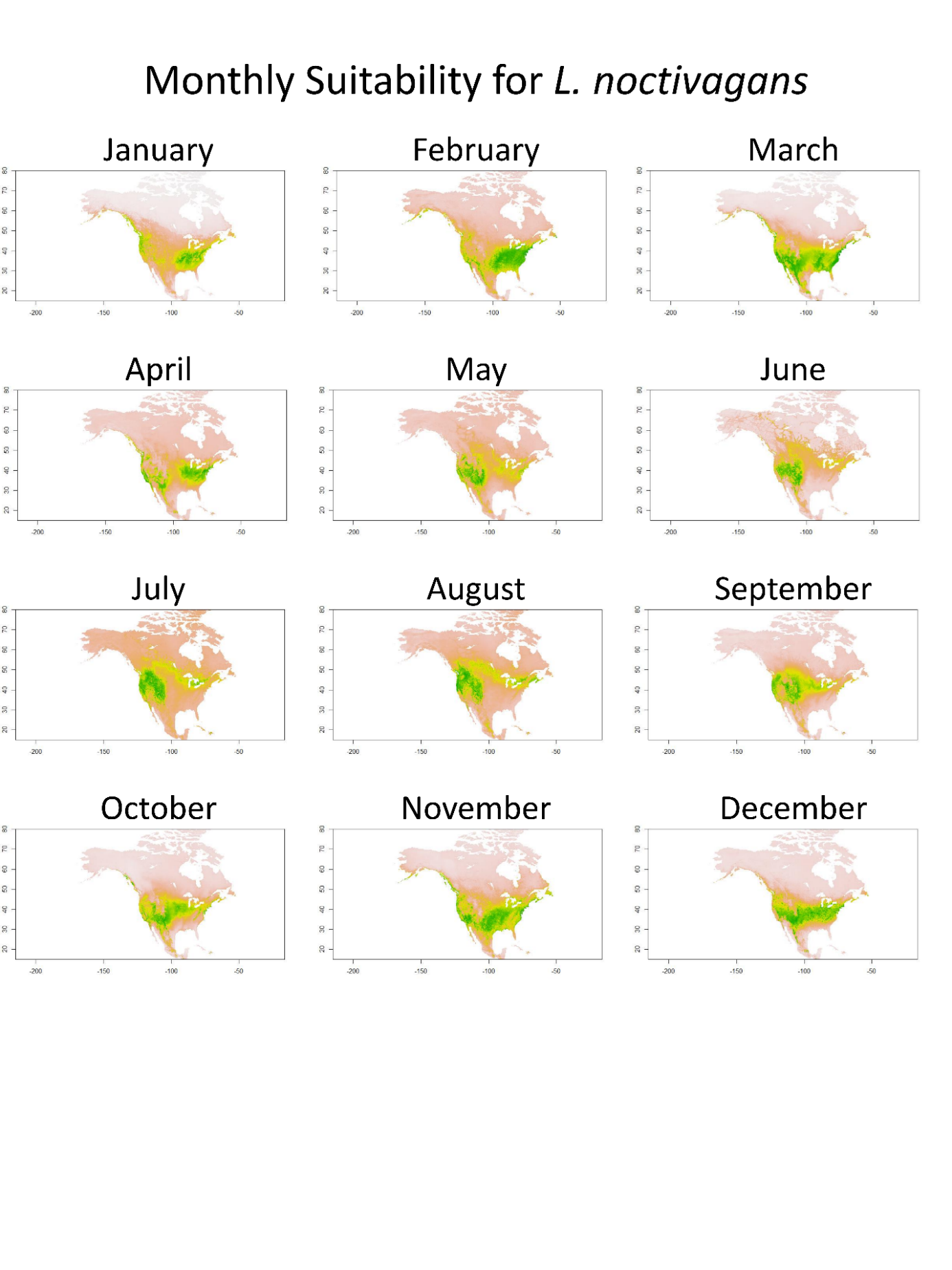

Supplement: Supplemental Information 5 — Monthly SDM from TSS weighted ensemble model for L. noctivagans across North America. Green equals higher suitability, while yellow/tan show lower suitability. [file peerj-09-11177-s005.png]

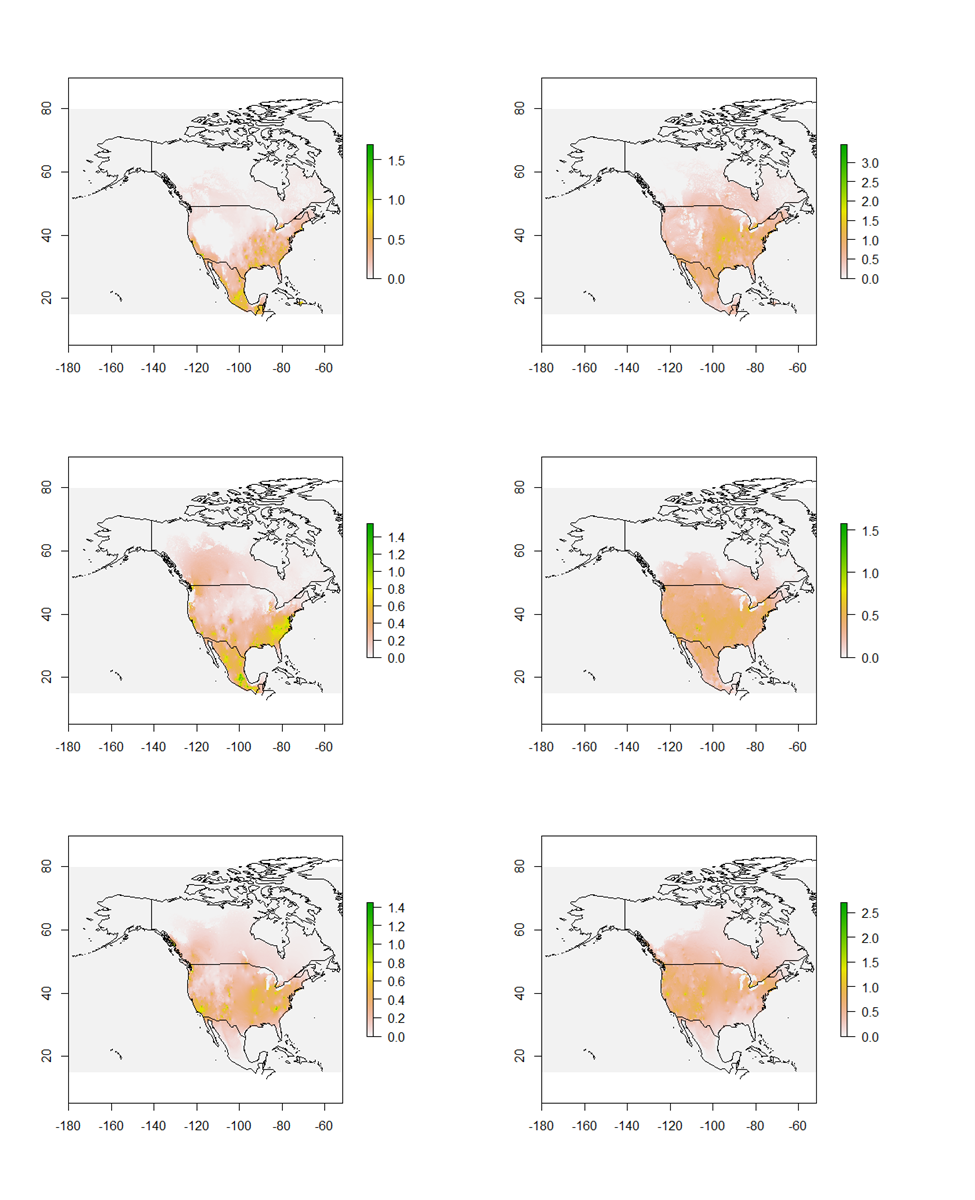

Supplement: Supplemental Information 6 — Circuitscape movement corridors for all three species (in order, L. borealis, L. cinereus, and L. noctivagans) for both spring (left) and fall (right). [file peerj-09-11177-s006.png]

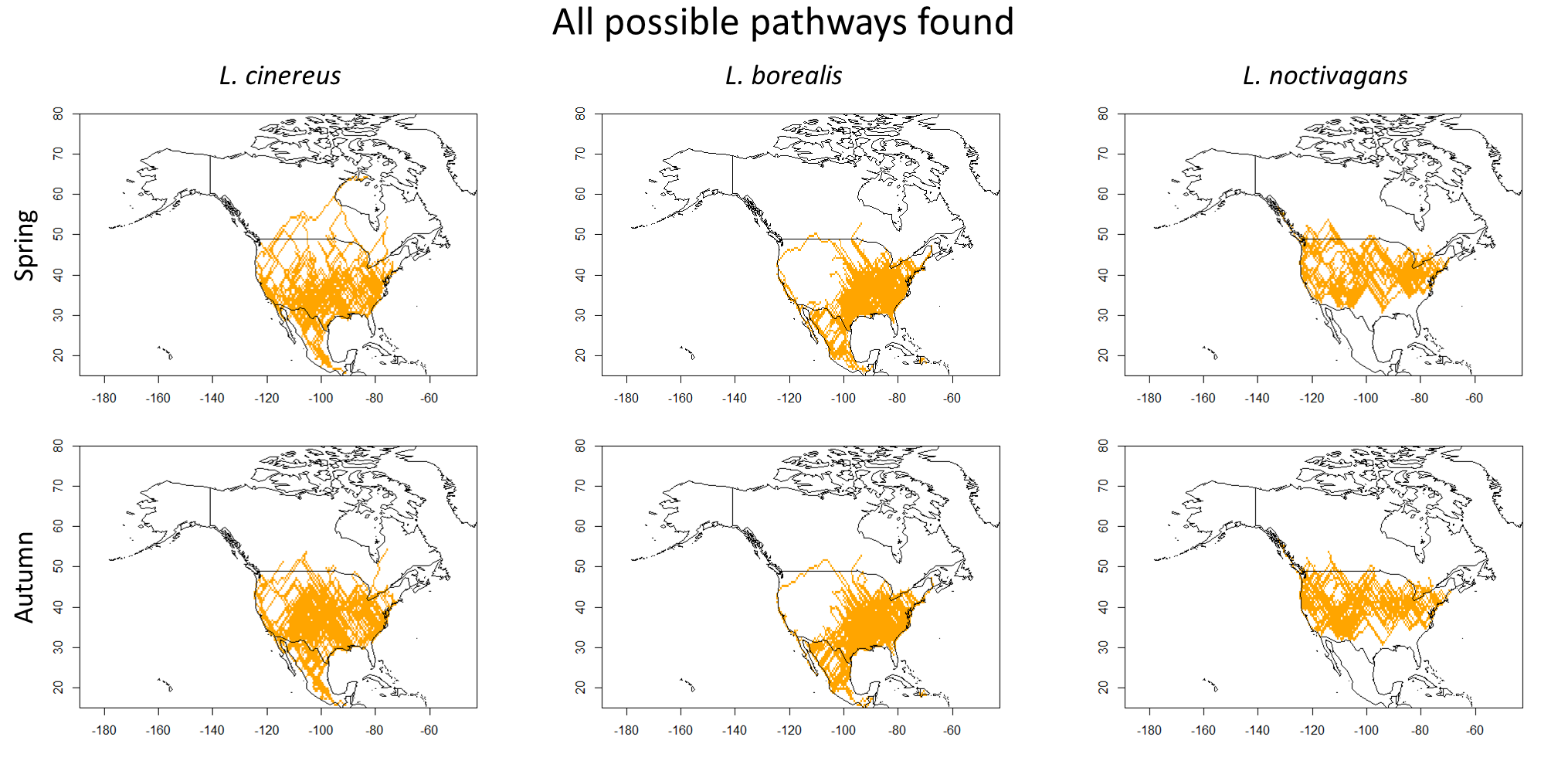

Supplement: Supplemental Information 7 — Map showing all possible paths found using least-cost-path analysis. While Fig. 4 shows the most likely paths, these maps use those and project all cells above 1. This allows for visualization of all possible paths found. [file peerj-09-11177-s007.png]
