# Supplementary material for: Predicting migration routes for three species of migratory bats using species distribution models
Source: PeerJ. 2021 Apr 16;9:e11177. doi: 10.7717/peerj.11177 (PMC8054759; doi:10.7717/peerj.11177)
Supplement: Supplementary file 3 [file peerj-09-11177-s008.docx]

		Random Forest											GLM											Maxent										
Month	Species	Prec	srad	tavg	tmax	tmin	vapr	wind	humam_inf	elev	NDVI	forest	Prec	srad	tavg	tmax	tmin	vapr	wind	humam_inf	elev	NDVI	forest	prec	srad	tavg	tmax	tmin	vapr	wind	human_ing	elev	NDVI	forest
apr	labo	10.38	9.74	10.82	11.08	12.33	13.04	8.55	7.70	10.34	7.56	4.75	2.80	0.99	1.13	1.15	1.10	0.38	1.18	1.64	2.91	0.71	2.21	1.8	4.2	0	8.1	75.6	2.9	0.2	1	2.9	3.1	0.3
aug	labo	15.26	22.42	24.31	24.99	31.18	35.50	15.56	20.97	19.30	21.14	11.98	2.29	0.73	0.40	0.40	0.40	3.97	1.56	3.37	1.19	1.40	0.11	2.3	10.5	0.9	5.7	41	17.6	1.8	8.5	3.4	6.4	2.2
dec	labo	3.68	5.06	3.99	4.31	4.40	5.05	1.71	3.09	4.37	3.67	1.65	2.39	0.77	1.78	1.76	1.80	0.10	1.42	1.70	2.54	1.60	1.15	2.7	24.4	0.9	6.4	13.9	11.2	7.3	9.6	5.1	16	2.4
feb	labo	4.16	5.07	5.16	4.73	5.16	4.94	3.48	5.60	3.62	4.02	2.57	1.38	1.18	0.92	0.90	0.96	2.94	3.65	2.97	2.08	1.38	2.91	3.3	4	0	14.2	54.4	2	3.5	12.2	1.3	3.1	2
jan	labo	6.00	5.97	6.26	6.39	6.64	6.94	5.70	6.11	6.47	5.60	3.57	0.94	1.43	0.08	0.07	0.11	3.22	2.05	0.99	4.36	0.83	0.93	0.9	0.7	0	0	77.5	7.6	0.7	4.7	0.5	7	0.5
jul	labo	20.13	23.67	30.92	24.66	34.38	39.53	15.17	20.81	19.36	17.90	9.46	2.11	0.34	0.18	0.17	0.17	4.60	2.00	2.77	1.84	1.36	0.51	4.3	7.3	17.4	5.3	42.6	3.6	4.6	1.1	5.9	0.9	7
jun	labo	19.53	16.17	22.38	22.96	25.81	28.87	12.18	16.76	16.03	15.93	6.20	1.37	2.03	1.70	1.72	1.67	2.81	0.37	4.07	0.09	2.24	0.93	2.5	3.3	0	8.1	74.6	2.2	0	4.2	2.2	1.1	1.8
mar	labo	5.55	6.10	6.30	6.44	6.53	7.27	4.80	5.56	5.54	5.67	3.81	1.82	1.03	0.32	0.30	0.35	0.92	2.00	1.27	1.73	0.10	0.58	2.2	0	0	7.3	79.4	0.7	2.3	5.2	0	0.2	2.5
may	labo	12.14	10.48	14.44	14.47	16.65	17.66	9.96	10.52	12.31	11.99	5.91	3.30	2.09	0.64	0.63	0.67	0.71	0.13	2.35	1.39	1.22	2.58	0.5	1.8	1	6.9	54.1	28.8	1.3	2.6	0	0.9	1.9
nov	labo	6.31	6.35	6.50	6.63	7.38	8.29	6.08	5.85	5.66	5.47	4.18	0.98	1.72	0.33	0.34	0.32	1.68	2.09	2.48	3.29	1.31	1.16	1.7	0	0	12.7	64.5	3.2	2.6	2.8	0.2	10.2	2.1
oct	labo	8.29	10.02	10.65	10.71	13.98	14.58	7.72	11.23	14.60	12.21	4.94	0.18	1.64	0.98	0.95	0.99	0.10	1.05	3.44	2.78	0.19	2.21	3.2	0	1.1	6.8	14.5	49.1	1.6	15.1	7.2	0.1	1.1
sep	labo	12.75	14.98	14.91	14.89	16.34	19.75	11.62	15.14	14.89	15.52	7.80	2.07	0.40	0.70	0.69	0.74	0.12	1.11	3.43	0.47	2.09	1.04	2.3	10.5	0.9	5.7	41	17.6	1.8	8.5	3.4	6.4	2.2
apr	laci	8.33	18.46	12.21	12.87	11.49	10.81	7.44	6.53	11.33	8.86	4.78	2.10	6.78	1.58	1.55	1.64	3.97	1.52	0.42	0.23	0.38	0.74	1.9	32.8	0	0.6	17.4	20.3	3.5	14.5	5.6	2.9	0.5
aug	laci	15.06	24.70	18.29	19.03	18.57	17.37	14.11	13.45	19.45	14.00	5.00	1.09	1.30	0.30	0.30	0.32	0.99	1.44	2.89	1.80	1.94	1.96	1.9	32.8	0	0.6	17.4	20.3	3.5	14.5	5.6	2.9	0.5
dec	laci	1.69	3.65	3.30	3.12	3.53	3.96	1.77	2.19	4.45	3.37	0.77	0.83	3.81	0.64	0.60	0.69	3.07	2.24	1.78	1.63	3.21	0.14	14.1	8.2	8.6	1.7	35.7	16	2.4	5.7	3.4	2.9	1.4
feb	laci	3.69	4.72	5.38	5.23	5.43	5.52	4.27	5.20	8.02	5.87	2.50	0.63	2.58	0.84	0.80	0.89	2.96	2.59	2.73	0.42	2.52	1.54	0	0	0	0	82.3	2.1	0.4	2.1	0.6	10.8	1.9
jan	laci	3.09	4.64	4.25	4.08	4.29	3.94	2.58	5.11	6.19	3.43	1.69	1.05	2.81	0.84	0.83	0.86	1.76	1.78	3.35	2.14	1.32	0.38	4	6.2	0	0.5	65.7	0	3.8	5.8	8.6	2.8	2.6
jul	laci	15.21	20.23	18.63	21.26	16.22	16.21	13.37	14.98	19.65	12.71	6.30	1.28	0.05	0.21	0.22	0.19	0.03	1.94	4.74	4.74	0.35	0.66	3.7	3.3	3.8	4.2	30.4	16.3	4.3	5.5	25.5	1.8	1.1
jun	laci	11.74	21.73	14.41	15.82	12.41	13.49	11.67	11.43	19.88	11.31	3.71	0.66	3.07	0.31	0.29	0.30	2.16	2.13	3.25	2.40	0.39	0.88	5.9	2.8	24.4	1	6.9	5.7	16.9	9.8	12.9	11.7	2.1
mar	laci	4.11	7.26	7.45	6.82	7.96	5.81	5.81	6.71	6.82	4.63	2.28	0.44	3.73	0.78	0.75	0.81	2.29	2.23	2.02	0.35	1.06	1.90	0.5	8.3	1.2	0	79	0.3	0	1.5	0	6	3.2
may	laci	13.44	19.92	14.24	14.64	12.74	13.02	10.78	9.89	15.50	11.34	3.30	1.79	4.55	1.03	1.04	0.99	3.19	2.15	0.93	3.36	1.64	0.95	3	19	4.8	2.7	31.4	10.4	1.8	2.8	6.7	9.6	7.7
nov	laci	2.72	5.47	6.95	5.19	5.66	5.61	3.35	3.15	7.98	3.85	1.91	1.73	6.01	0.97	1.05	0.90	4.25	2.00	1.33	1.13	1.20	1.02	5.6	0	1.4	3.1	65.6	4.2	0	7.7	0.3	9.8	2.2
oct	laci	10.22	19.19	12.19	12.14	13.18	11.39	8.53	7.70	11.70	11.81	3.88	2.06	4.33	0.01	0.07	0.03	1.50	1.03	0.46	1.12	0.98	0.65	5.3	16.8	0.3	11.5	44.5	8.1	0.1	3.3	7.5	0	2.5
sep	laci	15.36	18.99	15.90	14.70	14.91	14.36	12.31	11.25	14.15	11.49	3.88	0.50	2.55	0.24	0.25	0.24	4.13	1.36	1.54	2.64	1.65	0.10	1.9	32.8	0	0.6	17.4	20.3	3.5	14.5	5.6	2.9	0.5
apr	lano	5.82	12.84	12.23	11.04	9.68	8.62	6.87	6.14	7.29	5.31	3.13	3.51	3.11	0.72	0.74	0.71	1.53	0.61	1.32	1.20	0.43	0.32	6.2	7.7	27	8.8	46.4	0	0.5	0.1	1.5	1.5	0.2
aug	lano	14.94	22.74	20.06	18.65	15.44	14.40	13.29	11.83	20.74	10.74	6.71	1.97	5.08	0.68	0.73	0.64	1.31	3.07	2.34	1.39	1.02	0.28	5	22.8	27.4	3	15.3	10.1	3.5	4.7	4.4	2.4	1.4
dec	lano	1.90	3.01	2.89	2.83	2.67	2.25	2.52	2.01	3.31	2.40	1.32	1.33	1.67	1.39	1.38	1.40	2.47	0.53	0.42	0.13	1.11	0.70	0	26.9	59.7	0	0	1.4	0	0	0	11.9	0.1
feb	lano	3.32	3.53	3.37	3.35	3.36	2.98	3.10	3.24	2.72	3.57	1.17	1.00	0.64	0.10	0.09	0.12	3.00	0.64	2.20	1.38	0.09	0.49	7.8	14.7	28.9	1.2	16.5	16	1	5.4	5.8	2.4	0.2
jan	lano	3.97	3.78	4.15	4.14	4.27	3.82	2.76	3.38	3.46	3.02	2.10	3.58	1.34	1.09	1.10	1.08	1.85	0.14	0.91	0.66	1.82	0.50	0	11.2	34.5	0	0	16.3	0	26.3	0	0	11.8
jul	lano	11.12	18.19	12.64	13.11	12.68	15.14	12.66	11.33	19.20	10.96	6.03	1.48	4.97	0.89	0.85	0.95	1.56	1.27	2.14	4.48	1.59	0.06	1.6	21.7	0	18.1	16.8	12.2	1.9	17.3	7.9	0.8	1.6
jun	lano	10.95	22.26	14.62	16.13	15.06	15.28	9.26	10.03	21.99	10.32	5.96	0.82	5.61	2.58	2.58	2.60	2.06	0.36	1.57	3.65	2.76	0.86	3.2	18.5	0	18.2	16.2	11.7	1.2	18.9	7.4	0.9	3.7
mar	lano	3.26	4.34	4.19	4.06	3.99	3.99	2.20	4.86	4.10	3.07	1.45	2.05	1.64	0.60	0.60	0.61	2.09	0.10	0.42	0.60	1.71	0.59	0.9	8.3	0	0	78.7	0	0	2.4	7	2.2	0.4
may	lano	11.17	18.69	17.07	17.51	17.43	15.13	10.11	9.71	16.71	11.00	3.80	2.10	4.73	1.31	1.28	1.32	3.04	0.41	2.61	0.40	1.63	1.47	7.9	19.1	0	24.6	9.8	19.8	0.2	7.9	5.8	2.3	2.6
nov	lano	4.01	5.83	5.77	6.15	5.70	5.16	5.36	5.31	4.85	5.09	2.18	0.36	2.07	1.29	1.27	1.32	3.87	0.25	1.53	2.15	0.33	0.22	0.3	13.8	0	15.8	50.9	6.1	2	3.6	2.9	0.1	4.4
oct	lano	7.24	11.42	12.04	12.71	9.82	10.51	9.25	6.86	10.69	9.35	2.74	0.79	3.93	0.52	0.50	0.54	5.31	0.56	0.74	2.62	0.11	0.10	2.2	23	5.5	4	29.1	23.1	2.8	2.6	5.2	2	0.5
sep	lano	13.20	14.47	15.31	17.90	14.75	16.83	12.17	10.12	12.62	9.69	3.83	0.07	4.66	1.03	1.06	1.00	3.89	0.63	4.20	2.65	1.50	1.16	1	38.1	13.8	3.4	16.9	17.4	0.7	2.8	3.6	2	0.3
