# Supplementary material for: Predicting migration routes for three species of migratory bats using species distribution models
Source: PeerJ. 2021 Apr 16;9:e11177. doi: 10.7717/peerj.11177 (PMC8054759; doi:10.7717/peerj.11177)
Supplement: Supplementary file 4 [file peerj-09-11177-s009.docx]

Month	Speices	AUC_rf	AUC_gl	AUC_mx	AUC_bc	AUC_AUCw_en	AUC_tssw_en	tss_rf	tss_gl	tss_mx	tss_bc	tss_AUCw_en	tss_tssw_en	thr_AUC	thr_tss
apr	labo	0.51	0.86	0.91	0.75	0.93	0.98	0.79	0.68	0.79	0.79	0.79	0.85	0.24	0.14
aug	labo	0.71	0.82	0.87	0.85	0.93	0.95	0.84	0.61	0.64	0.59	0.74	0.84	0.09	0.07
dec	labo	0.7	0.89	0.86	0.55	0.97	0.98	0.88	0.74	0.83	0.76	0.87	0.91	0.04	0.02
feb	labo	0.5	0.91	0.89	0.74	0.96	0.98	0.78	0.78	0.83	0.82	0.83	0.83	0.08	0.05
jan	labo	0.56	0.92	0.92	0.8	0.93	0.98	0.81	0.72	0.75	0.72	0.75	0.85	0.28	0.18
jul	labo	0.64	0.85	0.85	0.73	0.91	0.94	0.8	0.62	0.68	0.55	0.71	0.79	0.17	0.11
jun	labo	0.62	0.85	0.86	0.82	0.92	0.95	0.81	0.65	0.71	0.61	0.71	0.8	0.14	0.1
mar	labo	0.5	0.93	0.95	0.77	0.94	0.99	0.8	0.71	0.79	0.71	0.78	0.88	0.28	0.17
may	labo	0.57	0.82	0.85	0.79	0.91	0.97	0.82	0.59	0.71	0.64	0.72	0.83	0.09	0.06
nov	labo	0.5	0.81	0.89	0.68	0.93	0.98	0.78	0.65	0.75	0.75	0.75	0.83	0.29	0.14
oct	labo	0.56	0.87	0.89	0.78	0.93	0.97	0.83	0.61	0.77	0.73	0.76	0.85	0.17	0.11
sep	labo	0.59	0.86	0.86	0.75	0.91	0.97	0.84	0.57	0.67	0.66	0.67	0.84	0.17	0.11
apr	laci	0.59	0.94	0.93	0.78	0.94	0.98	0.82	0.68	0.76	0.74	0.76	0.87	0.27	0.17
aug	laci	0.59	0.73	0.8	0.67	0.88	0.95	0.82	0.46	0.56	0.44	0.58	0.84	0.15	0.08
dec	laci	0.63	0.97	0.94	0.71	0.98	0.99	0.85	0.64	0.87	0.85	0.88	0.95	0.16	0.1
feb	laci	0.56	0.97	0.97	0.82	0.98	0.99	0.81	0.76	0.87	0.85	0.87	0.92	0.14	0.09
jan	laci	0.5	0.85	0.9	0.72	0.94	0.98	0.78	0.59	0.78	0.75	0.77	0.89	0.24	0.15
jul	laci	0.64	0.84	0.88	0.74	0.91	0.96	0.84	0.46	0.63	0.49	0.66	0.86	0.14	0.09
jun	laci	0.6	0.83	0.83	0.74	0.9	0.95	0.81	0.53	0.63	0.57	0.67	0.83	0.07	0.05
mar	laci	0.61	0.88	0.91	0.94	0.94	0.98	0.83	0.65	0.74	0.78	0.74	0.88	0.15	0.11
may	laci	0.53	0.82	0.86	0.7	0.92	0.96	0.8	0.63	0.72	0.64	0.72	0.81	0.11	0.07
nov	laci	0.51	0.79	0.9	0.53	0.96	0.98	0.81	0.65	0.78	0.72	0.79	0.92	0.09	0.04
oct	laci	0.52	0.86	0.87	0.73	0.91	0.97	0.79	0.58	0.66	0.56	0.66	0.82	0.15	0.09
sep	laci	0.51	0.79	0.85	0.74	0.88	0.96	0.79	0.52	0.6	0.54	0.59	0.83	0.2	0.12
apr	lano	0.54	0.75	0.89	0.79	0.92	0.97	0.8	0.52	0.74	0.64	0.74	0.85	0.24	0.14
aug	lano	0.65	0.84	0.86	0.85	0.93	0.96	0.84	0.55	0.68	0.62	0.75	0.84	0.11	0.08
dec	lano	0.5	0.6	0.81	0.55	0.87	0.95	0.78	0.47	0.68	0.68	0.68	0.78	0.43	0.13
feb	lano	0.5	0.85	0.84	0.5	0.89	0.95	0.77	0.61	0.61	0.66	0.63	0.79	0.11	0.05
jan	lano	0.5	0.73	0.78	0.64	0.89	0.96	0.83	0.58	0.68	0.66	0.68	0.83	0.15	0.08
jul	lano	0.55	0.87	0.89	0.78	0.92	0.97	0.8	0.56	0.73	0.66	0.72	0.84	0.12	0.08
jun	lano	0.6	0.77	0.88	0.71	0.93	0.97	0.83	0.47	0.67	0.54	0.71	0.85	0.18	0.13
mar	lano	0.5	0.82	0.85	0.63	0.9	0.96	0.77	0.6	0.68	0.71	0.67	0.8	0.08	0.04
may	lano	0.59	0.83	0.83	0.71	0.93	0.96	0.8	0.5	0.7	0.65	0.73	0.82	0.16	0.08
nov	lano	0.5	0.79	0.78	0.59	0.87	0.96	0.78	0.44	0.65	0.62	0.63	0.78	0.23	0.12
oct	lano	0.53	0.85	0.86	0.68	0.91	0.97	0.8	0.54	0.64	0.66	0.64	0.82	0.12	0.07
